# A Comparative Study of Airway Nerve Blocks and Atomized Lidocaine by the Laryngo-Tracheal Mucosal Atomization Device (LMA MADgic) Airway for Oral Awake Fiberoptic Intubation

**DOI:** 10.7759/cureus.15772

**Published:** 2021-06-20

**Authors:** Urvashi Yadav, Atit Kumar, Priya Gupta

**Affiliations:** 1 Anesthesiology, Uttar Pradesh University of Medical Sciences, Etawah, IND

**Keywords:** airway, conscious sedation, fiber optic technology, local anaesthesia, nerve block.

## Abstract

Background

Awake intubation is used most commonly in patients with a predicted difﬁcult airway. There are situations where the safest method to secure an airway is to place an endotracheal tube in an awake and spontaneously breathing patient. Our aim was to compare the two modalities, airway nerve blocks and atomized lidocaine by the Laryngo-Tracheal Mucosal Atomization Device (LMA MADgic)airway for awake fiberoptic intubation (AFOI).

Methods

A total of 50 patients with anticipated difficult airway requiring AFOI were randomly allocated into two groups. Group A received airway blocks (bilateral superior laryngeal and transtracheal recurrent laryngeal nerve) each with 2 ml of 2% lidocaine and group B received airway anesthesia through atomized lidocaine by LMA MADgic using 10 ml of 2% lidocaine. Fiberoptic guided orotracheal intubation was then performed in both the groups using LMA MADgic as the conduit. The primary outcome measured was intubation time and the secondary outcome included quality of intubation, hemodynamic variables, and any adverse events.

Results

The intubation time was found to be significantly lower in Group A (63.80±7.86 seconds) as compared to Group B (184.96±13.38 seconds) (p=0.0001). The ease of intubation, intubating condition, and patient comfort were better in patients who received airway blocks. Group B had an increased number of coughing/gagging episodes as compared with Group A. Between the two groups, group A showed better hemodynamics and fewer episodes of desaturation than group B.

Conclusion

Upper airway nerve blocks provide faster intubation, adequate airway anesthesia, and less patient discomfort to aid in AFOI in patients with anticipated difficult airway as compared to topical anesthesia using an atomizer.

## Introduction

Tracheal intubation is the key skill in the care of unconscious, anesthetized, or severely ill patients. Tracheal intubation is sometimes difficult and may end in many complications, the most serious being hypoxemic brain damage and death. Soft tissue damage is often caused by traumatic attempts at intubation [1]. AFOI is the gold standard within the management of patients with an anticipated difficult airway. It is essential to sufficiently anesthetize the upper airway and suppress the gag, swallow, and cough reflexes before awake fiberoptic bronchoscope (FOB)-guided intubation and thus ensure patient comfort [2]. This can be done in multiple ways, which can broadly be divided into two groups: (a) Topical administration of local anesthetic (LA) and (b) Blockade of neural supply to the oropharynx and larynx. Topical anesthesia of the airway can be done in the form of sprays, gargles, lozenges, nebulization, or impregnated swabs of local anesthetics, which causes less trauma to the oropharyngeal and laryngeal tissues as compared to nerve blocks [3]. There has been no study comparing the use of airway nerve blocks and the Laryngo-Tracheal Mucosal Atomization Device (LMA MADgic) airway technique for oral awake fiberoptic intubation. The LMA MADgic airway is an innovative and elegantly designed fiberoptic oral airway characterized by having a syringe port for topical anesthesia injection to anesthetize the vocal cords and laryngeal mucosa and an oxygen connector that permits passive oxygenation during fiberoptic intubation [4]. This study was conducted to assess and compare the efficacy of airway nerve blocks with atomized lidocaine by the LMA MADgic airway to achieve upper airway anesthesia for awake oral fiberoptic intubation.

## Materials and methods

This prospective, randomized controlled study was conducted in the anesthesia department of a tertiary care center after obtaining Institutional Ethics and Scientific Committee approval (UPUMS/DEAN/2020-21/E.C.NO:132/2018, dated 26-10-2020). It was registered prospectively with the Clinical Trial Registry of India (www.ctri.nic.in) and the registration number for this trial is CTRI/2019/11/021884.

Fifty patients belonging to the American Society of Anesthesiologist classification I-III above 18 years, identified to have difficulty in intubation by conventional laryngoscopy. We included patients with Mallampatti Grade Ⅲ and Ⅳ, thyromental distance ≤6, restricted neck movements or neck extension not allowed as in cervical spine patients who required awake fiberoptic intubation (AFOI). Exclusion criteria included the patients who denied participation, with restricted mouth opening, pregnant patients, mental retardation, known case of allergy to local anesthetic, coagulopathy, and patients on anticoagulants or antiplatelet. All patients were explained about the airway procedure and informed consent was obtained. All patients underwent detailed pre-anesthetic evaluation, including history regarding the illness, surgeries, and comorbidities. Airway examination (mouth opening, Mallampati grading, thyromental distance, and temporomandibular joint and neck mobility) was done in all patients, and the findings noted. All patients received tablet ranitidine 150 mg and alprazolam 0.5 mg orally one night before and in the morning two hours before surgery.

Before arriving in the operating room (OR), an intravenous (IV) cannula with 18 gauge secured and injection glycopyrrolate 15 µgmkg-1 was administered intravenously half an hour before shifting the patient to the OR. After taking the patient to the OR, standard monitoring devices, including non-invasive blood pressure (NIBP), electrocardiogram (ECG), and pulse oximetry were applied, and baseline parameters like heart rate, NIBP, and oxygen saturation (SpO2) were recorded. The patients were randomly allocated into two groups, A and B, using computer-generated tables of random numbers.

Group A (n=25) received two sprays of 2% lidocaine into the mouth to topicalize the oral cavity, tongue, and palate followed by a bilateral superior laryngeal nerve block using 2 ml of 2% lidocaine on each side and transtracheal instillation of 2 ml of 2% lidocaine.

**Figure 1:**
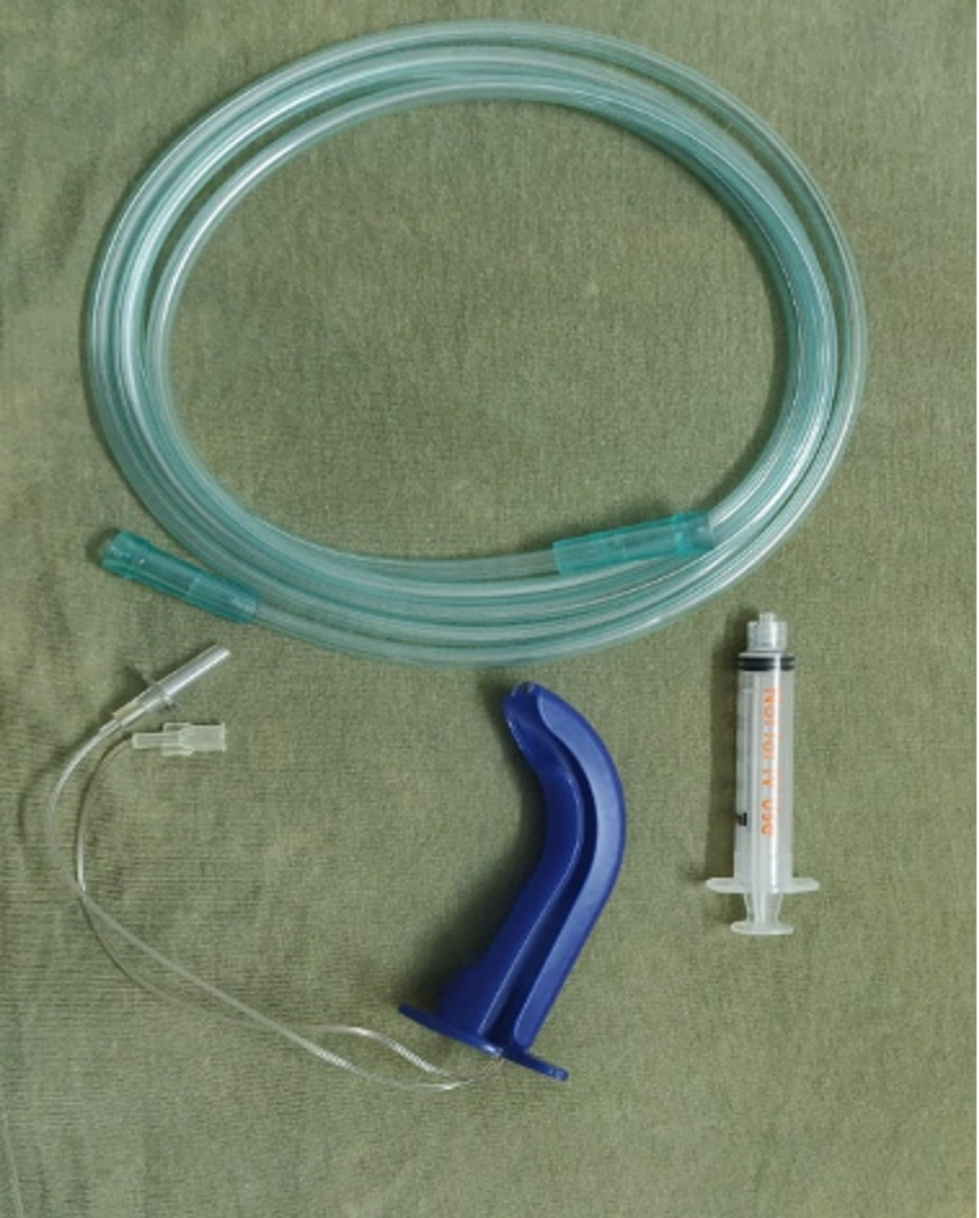
The LMA MADgic airway with syringe and oxygen tubing Laryngo-Tracheal Mucosal Atomization Device: LMA MADgic

Group B (n=25) received atomized lidocaine by the LMA MADgic airway (Figure 1) using 10 ml of 2% lidocaine.

The LMA MADgic airway was attached to oxygen tubing and a lidocaine-loaded syringe to obtain a fine mist directing towards the soft palate and posterior pharynx in a controlled fashion during the patient’s inspiration to topicalize the airway. The mouth, tongue, palate, and throat are first sprayed with topical anesthetic. Then it is inserted inside the oral cavity directed toward the glottic opening. Patients were asked to take full vital capacity breaths of atomized lidocaine containing oxygen to anesthetize the pharynx, glottis, and subglottic structures. Adequate local anesthesia was confirmed by hoarseness of voice in Group A patients and change of voice to low pitch and back of tongue becoming numb in Group B patients.

The oral fiberoptic bronchoscopy using the LMA MADgic airway as the working channel was performed after obtaining optimal topical anesthesia and adequate sedation in both groups. To ensure investigator blinding, atomization and blocks were administered by an independent anesthesiologist and two experienced consultant anesthetists (more than six years experience) clinically managed the trial. One mainly responsible for performing the AFOI procedure and the other mainly for observation and data collection. Both the patients and consultant anesthetists were blinded to group assignment. Oxygen was delivered at 4 L/min via nasal prongs placed in the nostril. All patients received the same drug delivery mode, with which dexmedetomidine was infused via a pressure‑driven syringe pump. All patients received IV infusion of dexmedetomidine 1 µgkg-1 over 10 minutes followed by a maintenance dose of 0.2-0.7 µgkg-1hr-1 to maintain a Ramsay sedation score-2 (1-Anxious, agitated or restless, 2-Cooperative, oriented and tranquil, 3-Responds to command only, 4-Brisk response to light glabellar tap or loud auditory stimulus, 5-Sluggish response to light glabellar tap or loud auditory stimulus, 6-No response to light glabellar tap or loud auditory stimulus). Ringer lactate 5-10 ml.kg−1.h−1 was administered simultaneously during the infusion of the study drug. After adequate sedation, a fiberoptic scope (Pentax FI 13 RBS, 4.2mm; Tokyo, Japan) loaded with an appropriate size endotracheal tube for patients was inserted orally using the LMA MADgic airway as a conduit into the hypopharynx in all patients. Once the position of the fiberscope in the trachea was confirmed, the airway was removed and the tracheal tube was railroaded and positioned approximately 3 cm above the carina and secured. After the confirmation of endotracheal tube position by auscultation and end-tidal CO2 (EtCO2), 2 mgkg-1 propofol IV and 0.1 mgkg-1 vecuronium IV were administered. Parameters including intubation time, quality of intubation in both the groups using cough and gag reflex score, grades of intubating condition, comfort score, and vital signs were recorded. To evaluate the quality of awake FOI, the levels of coughing and gagging during intubation and during the fixation of the ET were recorded separately by an independent anesthetist not involved with the intubation. The heart rate, systolic blood pressure, diastolic blood pressure, and oxygen saturation were noted prior to sedation, after Ramsay sedation score 2, during intubation, immediately after intubation, and 5 minutes after intubation. Hypotension was defined as systolic blood pressure (SBP) <80 mmHg, diastolic blood pressure (DBP) <50 mmHg, or an SBP decrease to ≥30% below baseline. Hypertension was defined as SBP >180 mmHg, DBP >100 mmHg, or an SBP increase to ≥30% above baseline. Bradycardia was defined as HR <50 beats/min or a decrease to ≥30% below baseline. Tachycardia was defined as HR >120 beats/min or an increase to ≥30% above baseline. Respiratory depression has been defined as respiratory rate <8 breaths/min or a fall to ≥25% below baseline. Hypoxia was defined as pulse oxygen saturation (SpO2) <94% or 10% lower than baseline. If any hypoxic episode occurred without an improvement via instructing to take deep breathes, infusion of the study drug was discontinued, and the patient was aroused.

Measuring variables

The primary outcome to be measured was intubation time. The intubation time (from inserting the fiberoptic scope into the nostril to confirmation of tracheal intubation with capnography) was measured. The quality of AFOI was measured by intubation score, cough and gag reflex, and comfort score.

The intubation score was measured by the patient's reaction to the placement of the fiberoptic scope and the tracheal tube on a 5‑point scale (1 = no reaction; 2 = slight grimacing; 3 = severe grimacing; 4 = verbal objection; and 5 = defensive movement of head, hands, or feet) [1].

Cough and gag reflex was measured as 1 = None, 2 = Minimal coughing and gagging, <3 times, 3 = Mild cough and gag lasting for <1 min, 4 = Persistent coughing and gaging) [1].

Every patient was asked to grade his/her comfort of the procedure (1 = Good, 2 = Moderately comfortable, 3 = Poor, uncomfortable) [1].

The changes in heart rate (HR), systolic and diastolic blood pressure, SpO2 during the procedure, and adverse effects if any were noted and compared.

After collection, data were analyzed by using Statistical Package for Social Sciences (SPSS) 16.0 version. The statistical analysis of quantitative data (Mean±SD) between the groups was done by the unpaired t-test. The statistical analysis of qualitative data (N%) between the groups was done by the chi-square test. P-value <0.05 was considered to be statistically significant.

## Results

**Figure 2:**
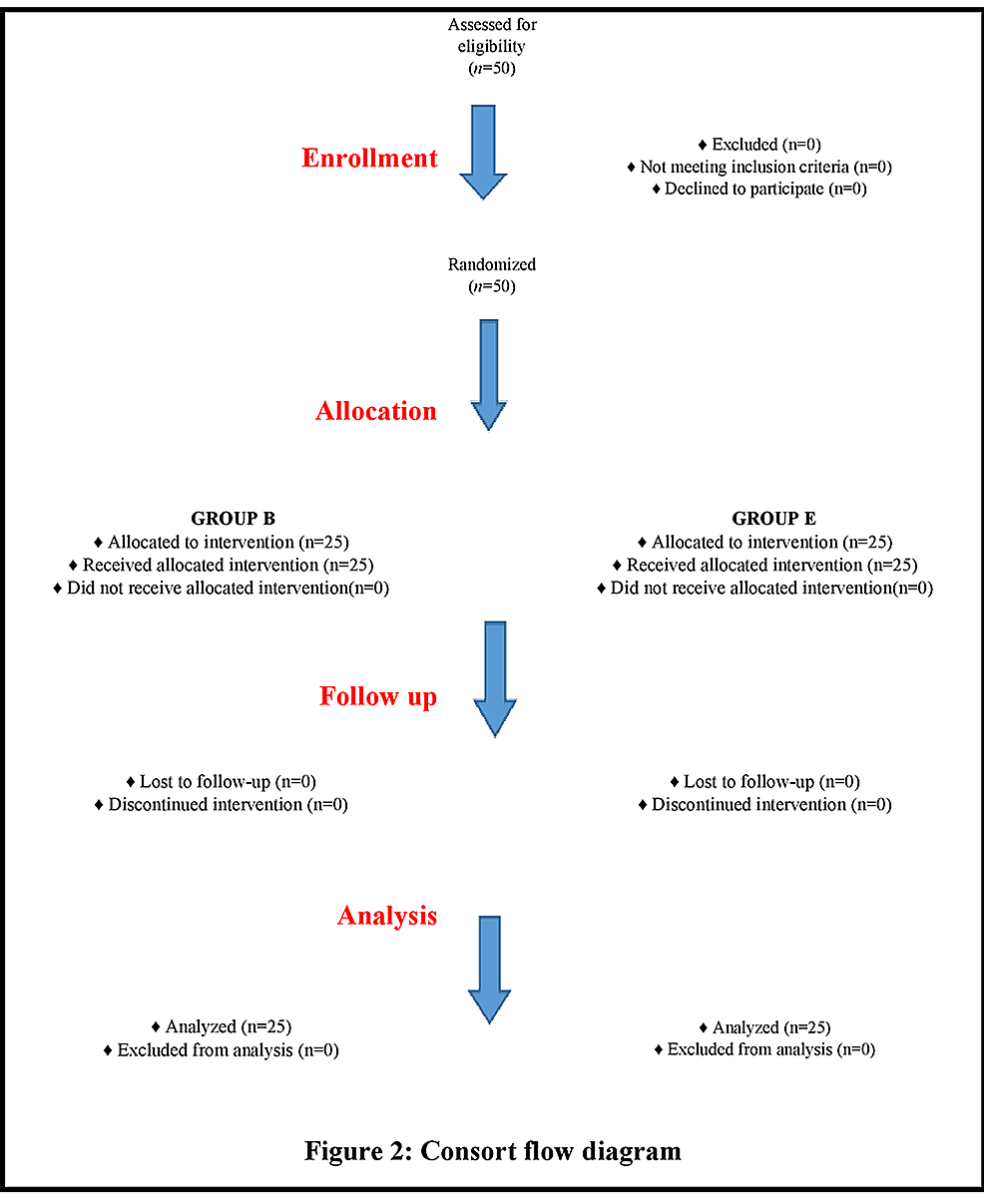
Consort flow diagram

All patients completed the study (Figure 2).

The demographic characteristics (age, gender, body mass index (BMI), and American Society of Anesthesiologists (ASA) physical status classification) were comparable between both groups (p>0.05) (Table 1). The groups were also comparable in terms of airway difficulty, as assessed by modified Mallampatti grade, mouth opening, and thyromental distance (Table 1).

**Table 1:** Demographic profile and Airway assessment of patients. SD=Standard deviation, ASA=American Society of Anesthesiologists Classification, BMI= Body mass index.

		Group A (n=25)	Group B (n=25)	P value
Age (yrs) (mean±SD)		35.44±8.29	34.44±8.89	0.68
Gender	Male	20	22	0.44
	Female	5	3	
BMI (kg.m^-2^) (mean±SD)		27.65±2.89	26.06±2.70	0.07
ASA Classification	ASA 1	17	16	0.76
	ASA 2	8	9	
Mouth Opening		2.88±0.21	2.86±0.22	0.75
Thyromental Distance		5.22±0.09	5.26±0.08	0.16
Mallampati Grade	Grade Ⅲ	19	18	0.74
	Grade Ⅳ	6	7	

The intubation time was found to be significantly lower in Group A (63.80±7.86 seconds) as compared to Group B (184.96±13.38 seconds) with p-value = 0.0001 (Table 2).

**Table 2:** Comparison of intubating conditions in both groups Cough and gag reflex (1 = None, 2 = Minimal coughing and gaging, <3 times, 3 = Mild cough and gag ≥3, 4 = Persistent coughing and gagging), Intubation score (1 = no reaction; 2 = slight grimacing; 3 = severe grimacing; 4 = verbal objection), Comfort score (1 = Good, 2 = Moderately comfortable, 3 = Poor, uncomfortable)

	Group A	Group B	P-value
Cough and gag reflex (1/2/3/4)	18/3/2/2	2/2/10/11	P=0.001
Intubation score (1/2/3/4)	22/3/0/0	0/6/19/0	P=0.001
Comfort score (1/2/3)	20/5/0	0/6/19	P=0.0001
Intubation time (Sec)	63.80±7.86	184.96±13.38	P= 0.0001

**Figure 3:**
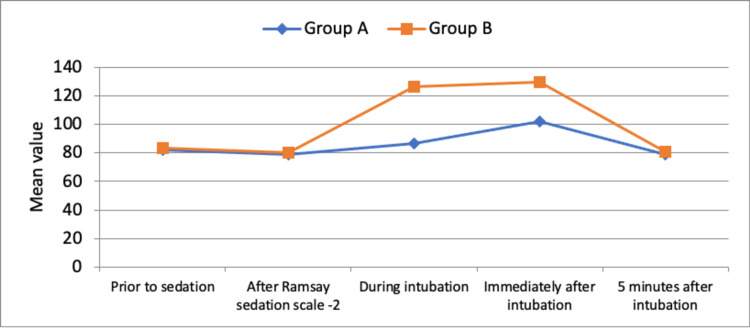
Comparison of changes in HR between the groups across the time period HR: heart rate

**Figure 4:**
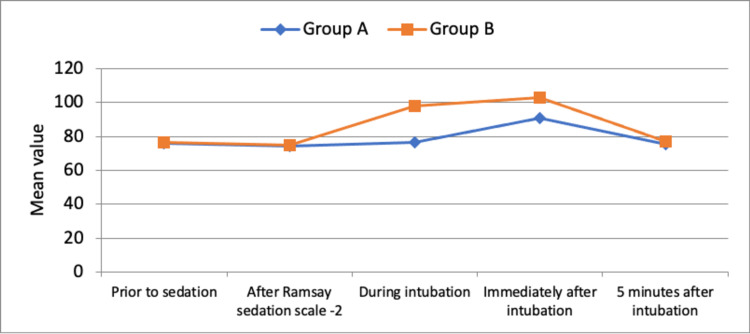
Comparison of changes in SBP between the groups across the time periods SBP: systolic blood pressure

**Figure 5:**
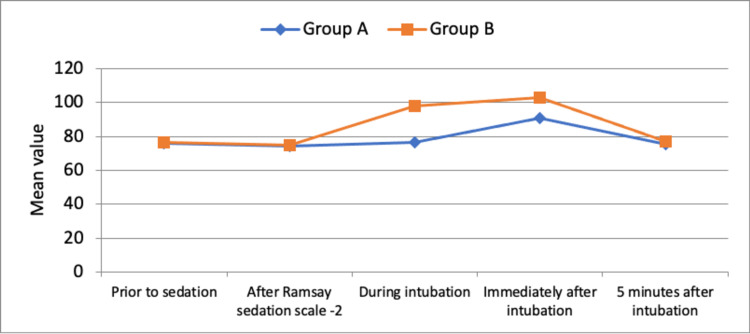
Comparison of changes in DBP between the groups across the time period DBP: diastolic blood pressure

**Figure 6:**
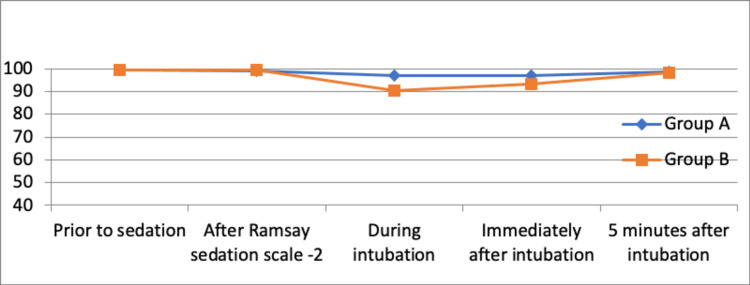
Comparison of changes in SpO2 between the groups across the time period SpO2: oxygen saturation

A significant number of patients experienced gagging and coughing during the procedure in atomization with a p-value of 0.001 (Table 2). The intubating conditions and comfort scores for Group A were better than those for Group B with a p-value of 0.001 and 0.0001, respectively (Table 2). Both the nerve block group and the atomization group had no statistically significant changes in heart rate, systolic blood pressure, diastolic blood pressure, and oxygen saturation prior to sedation and after Ramsay sedation scale-2. But there was a significant increase in HR (Figure 3), SBP (Figure 4), DBP (Figure 5), and change in SPO2 (Figure 6) in both the groups during intubation and immediately after intubation. However, these changes were transient and normalized after five minutes of fiberoptic intubation.

## Discussion

Awake intubation is commonly done in patients with a predicted difﬁcult airway [5]. There are situations where it is required to intubate an awake and spontaneously breathing patient [6]. Fiberoptic intubation can be performed nasally or orally in awake patients with topical or regional anesthesia alone, or in sedated or anesthetized patients. Patients with a large tongue, limited mouth opening, receding lower jaw, or tracheal deviation may benefit from a nasal approach, as well as circumstances where an unobstructed surgical field is required as in dental surgery [7]. If performed orally, several oropharyngeal airways are available to be used for the assistance of ﬁberoptic intubation such as Ovassapian, Williams, Berman, LMA MADgic, modiﬁed Guedel’s, and modiﬁed William’s airways [8].

There are several ways of anesthetizing the airway before performing awake FOB-guided intubation, such as nebulization, gargles, lozenges, sprays, and airway blocks, and LA through the working channel of FOB is usually used. LMA MADgic had been developed to facilitate a wider spread of local anesthetic than that obtained following spray and, consequently, may provide a similar or better quality of anesthesia for awake fiberoptic intubation. We compare the efficacy of atomization with an airway nerve block using the LMA MADgic airway.

In the present study, the time taken for intubation was significantly less in the nerve block group. It was similar to the findings of Gupta B et al., who reported an intubation time of 123 (46.7) seconds in a nerve block group and 200.4 (72.4) seconds in the nebulization group (p=0.047) [3]. Vasu BK et al. also concluded that the intubation time was significantly shorter in transtracheal topical anesthesia (48.5±38.6S) than in atomized local anesthetic (88.8±36.3 S) (p=0.019) [9]. Mathur PR et al. also found similar results in which mean intubation time was shorter in airway nerve blocks (115.2±14.7 s) as compared to lidocaine nebulization (214.0±22.2 s) [10]. It was also proven by Singh J et al. that the time taken to perform FOB-guided intubation was significantly lower in the nerve block group (90.2±11.7 s) as compared to the atomizer group (210.4±10.6 s) [11].

We observed that most patients in the nerve block group showed a significant reduction in the cough and gag reflex on passing the scope to the trachea as compared to the atomizer group. Twenty-eight percent (28%) of patients in Group A (7 out of 25) had an incidence of cough and gag reflex in comparison to 92% (23 out of 25) in Group B (p=0.001). Topical anesthesia was not efficient enough in the atomization group; it may be due to the raining-down effect of local anesthetic into the trachea during atomization. This might have been the reason for less comfort observed in the patients in the atomization group. Gupta B et al. also compared two methods of anesthetizing the airway for awake fiberoptic tracheal intubation and found that a significant number of patients experienced gagging and coughing during the procedure in ultrasonic nebulization as compared with airway nerve blocks (p=0.004) [3]. Similar results were obtained in studies done by Singh J et al., who reported that atomized local anesthesia had higher cough and gag scores than airway nerve blocks with a p-value=0.006 [11]. The present study coincides with Vasu BK et al. in which Group A (atomized lignocaine) had higher cough and gag scores compared to group T (transtracheal injection) (p=0.001) [9], and similarly, Chandra A et al. found that the number of coughs in Group I (4±0.98) was significantly lower than in Group II (4.9±1.24, p<0.05) [12].

In the present study, the intubating conditions were better in Group A as compared with Group B. Similarly, Mathur PR et al. showed better intubating conditions in patients who received bilateral superior laryngeal and transtracheal recurrent laryngeal nerve blocks (each with 2 ml of 2% lidocaine) compared to patients who received jet nebulization (p=0.001) [10]. Gupta B et al. performed a randomized control study comparing airway nerve blocks with nebulized lidocaine using an ultrasonic nebulizer [3]. They did not find any significant difference regarding the intubating conditions (p=0.315).

Patient comfort score was higher in the nerve block group in the present study. This can be attributed to the deposition of local anesthetic in the vicinity of the nerves. However, during atomization, the local anesthetic is deposited over the mucosa, i.e., away from the nerves. Mathur PR et al. found that patient comfort during intubation, as assessed by cough severity (p=0.001) and intubation comfort scores (p=0.012), was higher in Group B than in Group N [10]. These findings are supported by Gupta B et al [3] who showed that patient comfort was better in the nerve blocks group as compared with the nebulization group.

Both the nerve block group and the atomization group had no statistically significant changes in heart rate, systolic blood pressure, diastolic blood pressure, and oxygen saturation prior to sedation and after Ramsay sedation scale 2 but there was an increase in heart rate, systolic blood pressure, and diastolic blood pressure in both the groups, during and immediately after intubation probably due to sympathetic stimulation after the tip of the scope passed beyond the vocal cord up until the carina is visualized. However, these changes were transient and normalized within five minutes after fiberoptic intubation. Between the two groups, the atomization group had a larger increase in SBP and DBP as compared to the nerve block group. Similarly, a study done by Kundra P et al. observed a progressive increase in heart rate and mean arterial blood pressure in all patients from the beginning of the procedure, but the rise in the nebulization group was greater (p<0.05) and also lasted longer than in the block group (p<0.05), with patients belonging to the block group demonstrating considerable hemodynamic stability [13].

## Conclusions

When compared to topical anesthetic using an atomizer, airway nerve blocks (bilateral superior laryngeal and transtracheal recurrent laryngeal) provide faster intubation, appropriate airway anesthetic, and less patient discomfort during AFOI in patients with a potentially difficult airway. Our limitation was the small sample size and a study with larger sample size is required for validation of results. The various scores used for assessment are based on the subjective responses of the individual, which could be variable and cannot be standardized. Third, we did not study the long-term effects of these blocks so we cannot prove the long-term safety of these blocks.
